# Future plans of veterinary graduates in Germany in 2023: an online survey

**DOI:** 10.3389/fvets.2025.1652931

**Published:** 2025-09-08

**Authors:** Mira Elisabeth Riemann, Martina Hoedemaker, Katharina Charlotte Jensen

**Affiliations:** ^1^Clinic for Cattle, Clinical Centre for Farm Animals, University of Veterinary Medicine Hannover, Foundation, Hannover, Germany; ^2^School of Veterinary Medicine, Institute for Veterinary Epidemiology and Biostatistics, Freie Universität Berlin, Berlin, Germany

**Keywords:** shortage of veterinarians, survey, veterinary graduates, influence on future planning, education, admissions

## Abstract

**Introduction:**

In Germany, as in other countries, a shortage of veterinarians is threatening the health and welfare of pets and livestock. Against this background, the aim of this study was to find out what plans veterinary graduates have after their studies, what field they want to work in and whether their career aspirations change during their studies. In addition, it was investigated whether the factors of gender, origin (urban/rural), and animal ownership before studying were associated with future planning/decision-making.

**Materials and methods:**

With the help of an online survey, German graduates of veterinary medicine from the class of 2023 were interviewed. Responses from 157 people were analyzed, which corresponds to a response rate of around 17%.

**Results:**

The results of this study show that almost half of the participants (*n* = 76; 48%) would like to work in a practice directly after graduation and 63% of respondents (*n* = 98) saw themselves working in a practice in 5 years’ time. The field of small animal medicine was the most strongly represented, followed by equine medicine. Only 42% of participants (*n* = 66) saw themselves in the same veterinary field in the future as they had aspired to before their studies. Neither gender nor origin were associated with future planning. However, the husbandry of certain animal species before studying was significantly associated with the field in which the respondents wanted to work later.

**Discussion:**

These results provide an initial basis for estimating how many veterinarians enter the respective fields and should be taken into account when discussing changing the admission requirements of universities of veterinary medicine to attract more young people to a certain animal field or a certain region of Germany.

## 1 Introduction

In many countries, there is a shortage of veterinarians, and a lot of practices and clinics have difficulties finding and retaining staff (1–3). Rural areas are particularly affected. In a survey in 2020, the Federation of Veterinarians in Europe (FVE) found that there was already a shortage of veterinarians in rural areas in almost 80% of European countries and in the upcoming years the situation would worsen in the remaining European countries (4). As early as 2006, Prince et al. predicted that there would not be enough veterinarians for farm animals in the following 12 years (from 2004 to 2016) in Canada and the USA (5). Currently, a shortage of (livestock) veterinarians is predicted for the future in the USA, Europe, in particular Switzerland and Bavaria, Germany (1, 4, 6–9). This shortage of veterinarians carries the risk that animal care will no longer be guaranteed. In Germany, there is already an emergency service crisis in the small animal sector, which means that sometimes patient owners must travel long distances with their ill or injured animals and have long waiting times (10). The consequences of a lack of veterinary care not only affect animal health and welfare, but also human health (11). Among other things, veterinarians are responsible for food hygiene/safety, and they play a key role in containing infectious diseases and preventing possible consequences for humans (12).

The future generations of veterinarians will contribute significantly to counteracting the shortage of veterinarians. Currently in Germany, more people apply to study veterinary medicine than there are places available (13). Therefore, one way to address the shortage of veterinarians is to offer more study places at the universities (14, 15). However, first it would make sense to find out how many graduates go into practice after graduation and in which type of veterinary practices they would prefer to work in. In Germany, reliable data for this are still lacking. The only German studies known to us focused on farm animal medicine and not on all animal species (16, 17). Although studies from other countries have also looked at other animal species (18–21), the results are not fully transferable to the German situation. In addition, a non-negligible portion of veterinarians work in areas outside of curative practice (at least 20% of working veterinarians in Germany (22), for example, are employed in research, industry, or in various authorities). These fields of work have been neglected in previous studies (18, 19, 23).

Based on this background, the aim of this study was to find out how many graduates would like to work in practices directly after their studies and where they see themselves in 5 years’ time. Particular attention was paid to how strongly the veterinary fields were represented and whether students changed this decision during their studies. In addition, it was investigated whether demographic factors such as gender, origin (urban/rural), and keeping animals before studying had an influence on their later career decision.

## 2 Materials and methods

First, hypotheses were defined and a literature review was conducted. Based on this, a questionnaire was created and deployed using LimeSurvey Version 3.23.1 + 200,825 (LimeSurvey GmbH, Hamburg, Germany). In February 2023, a pre-test was carried out to improve the quality of the survey. People from different groups (professors, students, veterinarians, and non-veterinarians) checked on comprehensibility and logic, detected errors and established the time needed to finish the survey. As the survey was well received, only minor changes were made. The survey was then activated and distributed in April 2023. The target group of the survey was the 2023 graduates of all five German educational institutions for veterinary medicine. They finished their studies between February and April, depending on the university. The graduates were invited to take part in the survey via a link sent to their university email addresses by the dean’s office of the universities and in some universities also by the German Veterinary Students Association (Bundesverband der Veterinärstudierenden in Deutschland e. V.). The invitation to participate was also issued by the Association of Employed Veterinarians (Bund angestellter Tierärzte e. V.), the respective semester spokespersons, and via social media (Instagram, Facebook). The survey was open until June 22, 2023.

The study was approved by the Chairman of the Commission for Research Ethics of the University of Veterinary Medicine Hannover, Foundation, Hannover, Germany (No. 25-PK-02) and conducted in compliance with German and European data protection regulations. The informed consent was embedded in the survey, which the participants had to agree to on the landing page.

### 2.1 The survey

In total, the questionnaire consisted of 64 questions, although not every participant had to answer every question, as 21 of them were conditional. The 64 questions included 10 open questions with free text answers, two multiple-choice questions (MC), 34 single-choice questions (SC), and 18 questions with different scales (e.g., yes/no or five-point Likert scales). The MC-questions and 19 of the SC-questions provided free text options to explain answers that did not match the given answer options. There were 20 mandatory questions that had to be answered to be able to continue with the survey, but the ‘no answer’ option was offered.

In terms of content, the questionnaire was divided into four sections. In the first section (14 questions), the participants were able to provide information about their age, gender, and origin as well as about the animals they had kept before studying. In the second section (seven questions), the focus of the questions was on the time before studying, e.g., in which field the respondents had wanted to work in before studying. The largest part of the survey was the third section with 29 questions. In this section, questions were asked about their veterinary medical studies, e.g., which university the participants were studying at. As the practical training of the graduates was influenced by the COVID-19 pandemic, questions on replacement programs and restrictions in practical training were included using a five-point Likert scale (‘I agree’ to ‘I disagree’). The last part (14 questions) focused on the graduates’ plans after their studies. They were asked to provide information about their plans after graduation and where they saw themselves in 5 years.

### 2.2 Evaluation and statistical analysis

The statistical analysis was carried out using the SAS Enterprise Guide (version 7.15). Only responses from people who had completed their studies in 2023, who had studied predominantly in Germany and who had answered the survey up to at least page 10 (of a total of 12) were evaluated. Categorical information (gender, origin, etc.) and the graduates’ future plans were analyzed descriptively using frequency tables and bar charts. The chi^2^ test, Fisher’s exact test, and the comparison between actual and expected frequencies were used to find out whether there was a significant difference between the variables under consideration. A significant difference was assumed from a value of *p* < 0.05. The change in decision between the time before graduation, immediately after graduation, and in 5 years’ time was depicted using a Sankey plot. This was implemented in R (version 4.4.0 ‘puppy cup’) with the packages readxl, dplyr, tidyverse, ggplot2, and ggsankey (24–28). As some questions had not been answered by all participants, the frequencies varied depending on the number of respondents.

New variables were transformed for the Sankey plot and the cross tables. The variable ‘future plans in 5 years’ is a combination of two questions: ‘Where would you like to be working in 5 years?’ (clinic/practice/non-curative (not working in practice, e.g., research or public health)/I do not know yet/other) and the question ‘In which animal field would you like to work in 5 years?’ (pets/ruminants/horses/pigs/poultry/mixed with cattle/mixed without cattle/exotic animals/other). The second question was only displayed if clinic or practice had been chosen in the first question. Finally, the new variable ‘future plans in 5 years’ contained the options I do not know (all answers with ‘I do not know yet’, ‘I have no plans yet’, and ‘no answer’), non-curative, pets (pets and exotic animals), horses, farm animals (poultry, pigs, and ruminants) and mixed. Respondents who marked ‘other’ for one of the questions were sorted into one of the existing categories according to the free text information. The variable ‘future plans directly after graduation’ was combined in the same way. For this variable, there were three additional options: timeout, internship, and dissertation. In Germany, veterinary students do not write a thesis within their studies but can write a doctoral thesis independently from the veterinary curriculum before or mostly after graduating. This dissertation corresponds rather to the scope of a PhD thesis than to a Master’s thesis. However, doctoral students may write their thesis while working at the university or in a practice. Our questionnaire did not include whether the doctoral students planned to work practically alongside their doctoral thesis. Five people indicated in the ‘other’ field that they would like to start working on a doctoral thesis directly after their studies while working at a practice or clinic. Those respondents were sorted to the option ‘practice’ or ‘clinic’. The animal field for those people was unknown.

One aim of this study was to find out which factors were associated with future plans after graduation and in 5 years’ time. The focus here was on four different animal fields (pets, horses, farm animals and mixed) and the non-curative field. Thus, the variables ‘I do not know’ and ‘other’ were not used for the calculation of chi^2^, Fisher’s exact test, and the expected frequencies.

For the question ‘How big was the place where you mainly grew up?’, the two categories 5,000–20,000 inhabitants and <5,000 inhabitants were grouped under rural and the other two categories (>100,000 inhabitants and 20,000–100,000 inhabitants) were grouped under urban. The answers to the question ‘Did you have pets/animals at home before your studies?’ were also summarized for the cross tables. The answers dog/cat, small mammals (e.g., rabbits), pet birds, and reptiles/exotic animals were summarized as the new answer option pets. The answer options cattle, sheep/goats, pigs, and poultry were grouped together as farm animals. As this question was a multiple-choice question, a further variable ‘just pets’ was created.

## 3 Results

### 3.1 Participants

A total of 311 people opened up the link to the survey. Due to the widespread invitation to participate, 142 people who were not part of the target population (veterinary medical students graduating in 2023) took part in the survey and answered at least some of the questions. Those respondents as well as 11 participants who answered fewer than 10 of 12 pages and one person who did not study in Germany were excluded. The response rate was 17% (Table 1). A homogeneous distribution between the universities was achieved.

**Table 1:** Distribution of participants in a survey conducted among German veterinary graduates from the class of 2023 across the five German veterinary schools.

Veterinary schools	Participants	Percentage of participants	Graduates of 2023*	Percentage participants of graduates 2023
Freie Universität Berlin	21	13	147	14
Justus Liebig University Gießen	23	15	179	13
Ludwig-Maximilians-Universität München	48	30	253	19
University of Veterinary Medicine Hannover, Foundation	35	22	238	15
Leipzig University	30	19	137	22
Total	157	100	944	17

* Status of September 4, 2023.

Of the participants, 90% (*n* = 141) identified themselves as female, 10% (*n* = 15) as male and none as any other gender. One person did not answer this question. The median age of the participants at the time of the survey was 26. Over a third (38%, *n* = 59) stated that they had grown up in a village with fewer than 5,000 inhabitants. Around a quarter (26%, *n* = 39) of the participants originated from a large city with over 100,000 inhabitants. Just under a fifth (19%, *n* = 30) of respondents had grown up in a city with 20,000–100,000 inhabitants and 17% (*n* = 27) in a small town with 5,000–20,000 inhabitants. Two people did not answer this question.

### 3.2 Plans after graduation and in 5 years’ time

Table 2 shows the veterinary field in which the respondents would like to work directly after graduation and in 5 years’ time.

**Table 2:** Graduates’ future plans directly after graduation and in 5 years’ time from a survey conducted among German veterinary graduates from the class of 2023.

Future plans	Directly after graduation		In 5 years’ time	
	*n*	%	*n*	%
Clinic	30	19	42	27
Practice	39	25	56	36
Internship	7	4	n.a.*	n.a.*
Dissertation	54	34	n.a.*	n.a.*
Non-curative	6	4	17	11
I do not have any plans yet/I do not know yet	7	4	40	25
Timeout (e.g., going abroad)	12	8	n.a.*	n.a.*
No answer	1	1	1	1
Other	1	1	1	1
Total	157	100	157	100

n.a. * = not available; this answer option was not available for the question on plans in 5 years’ time.

Nearly half (48%, *n* = 76) of the participants wanted to work in a practice directly after their graduation (Table 2). Compared to the time directly after graduation, 5 years later 15% more participants (63%, *n* = 98) saw themselves in a practice. This increase could be attributed to the fact that some would like to write a doctoral thesis first and before going into practice. Only 12% of participants (*n* = 19) did not yet know what they would like to do directly after their studies, or were planning to take a break (e.g., going abroad).

The respondents who indicated that they would like to work in a practice directly after their studies (clinic, practice, internship) or in 5 years (clinic, practice) were asked to specify the animal field in which they would like to work (Table 3).

**Table 3:** Animal field in which graduates who want to work in a practice would like to work in directly after graduation and in 5 years’ time from a survey conducted among German veterinary graduates from the class of 2023.

Animal field	Directly after graduation			In 5 years’ time		
	*n*	%		*n*	%	
		of participants in practice	of all participants		of participants in practice	of all participants
Pets	36	47	23	43	44	27
Ruminants	7	9	4	7	7	4
Horses	14	18	9	20	20	13
Pigs	2	3	1	4	4	3
Poultry	1	1	1	1	1	1
Mixed	8	10	5	19	19	12
Exotic animals	1	1	1	3	3	2
No answer/other	7	9	4	1	1	1
Total	76	100	48	98	100	63

As can be seen in Table 3, the field pets was most strongly represented among those who wanted to work in a practice, both directly after graduation and 5 years later. The equine field remained roughly constant. It is interesting that if they were aiming to work in a mixed practice, the participants preferred a mixed practice with a ruminant component (9%, *n* = 7, or 17%, *n* = 17) rather than a mixed practice without a ruminant component (1%, *n* = 1, or 2%, *n* = 2). Working with pigs, poultry, and exotic animals were the least popular both directly after graduation and in 5 years’ time.

**Figure 1:**
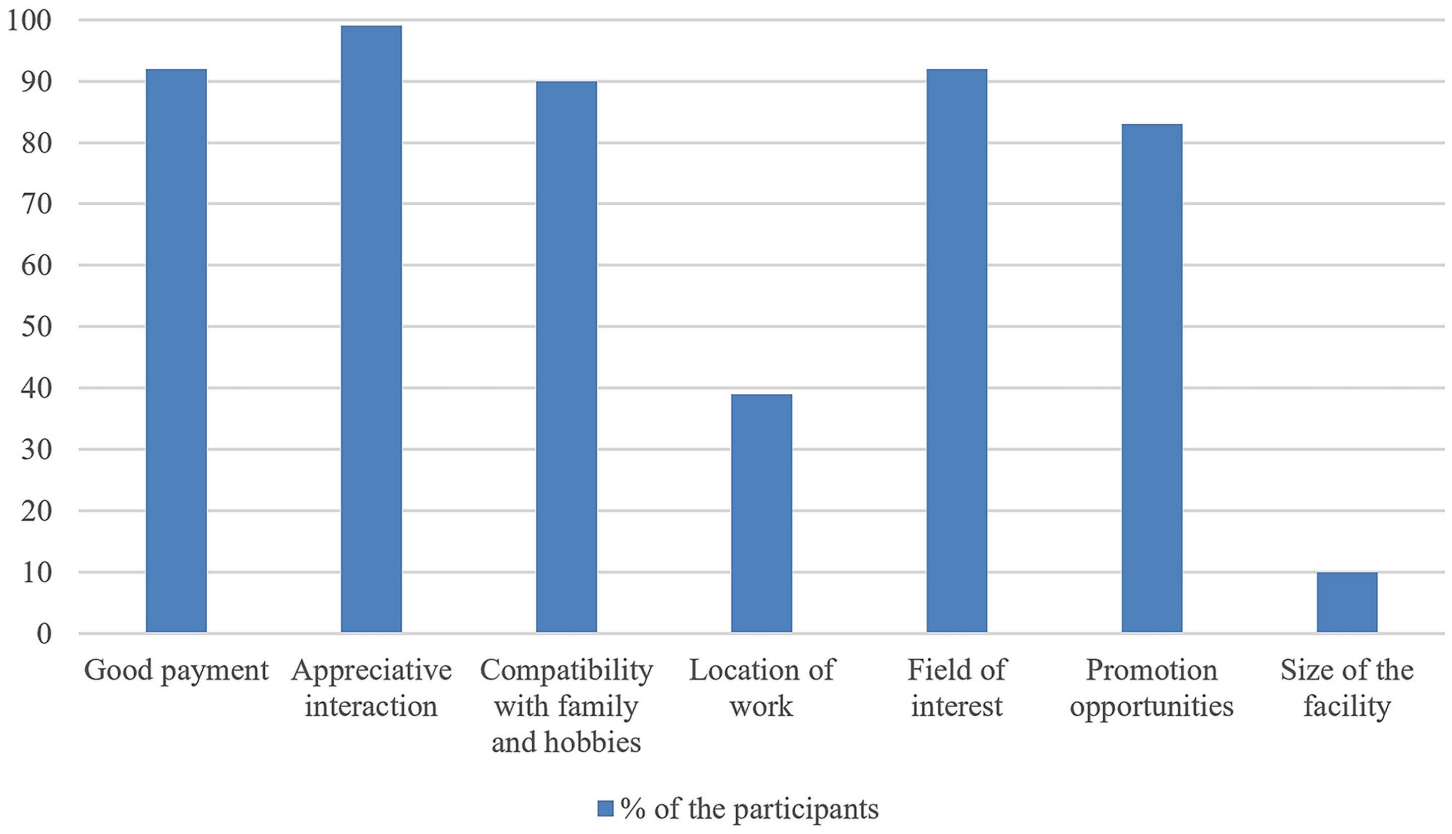
Different factors the participants considered either ‘particularly important’ or ‘important’ for their future job from a survey of German veterinary graduates from the class of 2023.

When asked about whether they wanted to work in an urban or rural area, 37% (*n* = 58) of the respondents stated that they preferred a rural area and 15% of them (*n* = 24) preferred to work in a city. Interestingly, 47% of the participants (*n* = 73) had no preference but agreed to the statement ‘The main thing is that I’m happy with the job’. Figure 1 shows the importance of different factors for the job search of graduates. The geographic location was only considered by 39% of the participants as important.

### 3.3 Changes in future plans

To assess whether the choice of veterinary field had changed during their studies, the participants were asked in which field they had imagined working in later before their studies.

**Figure 2:**
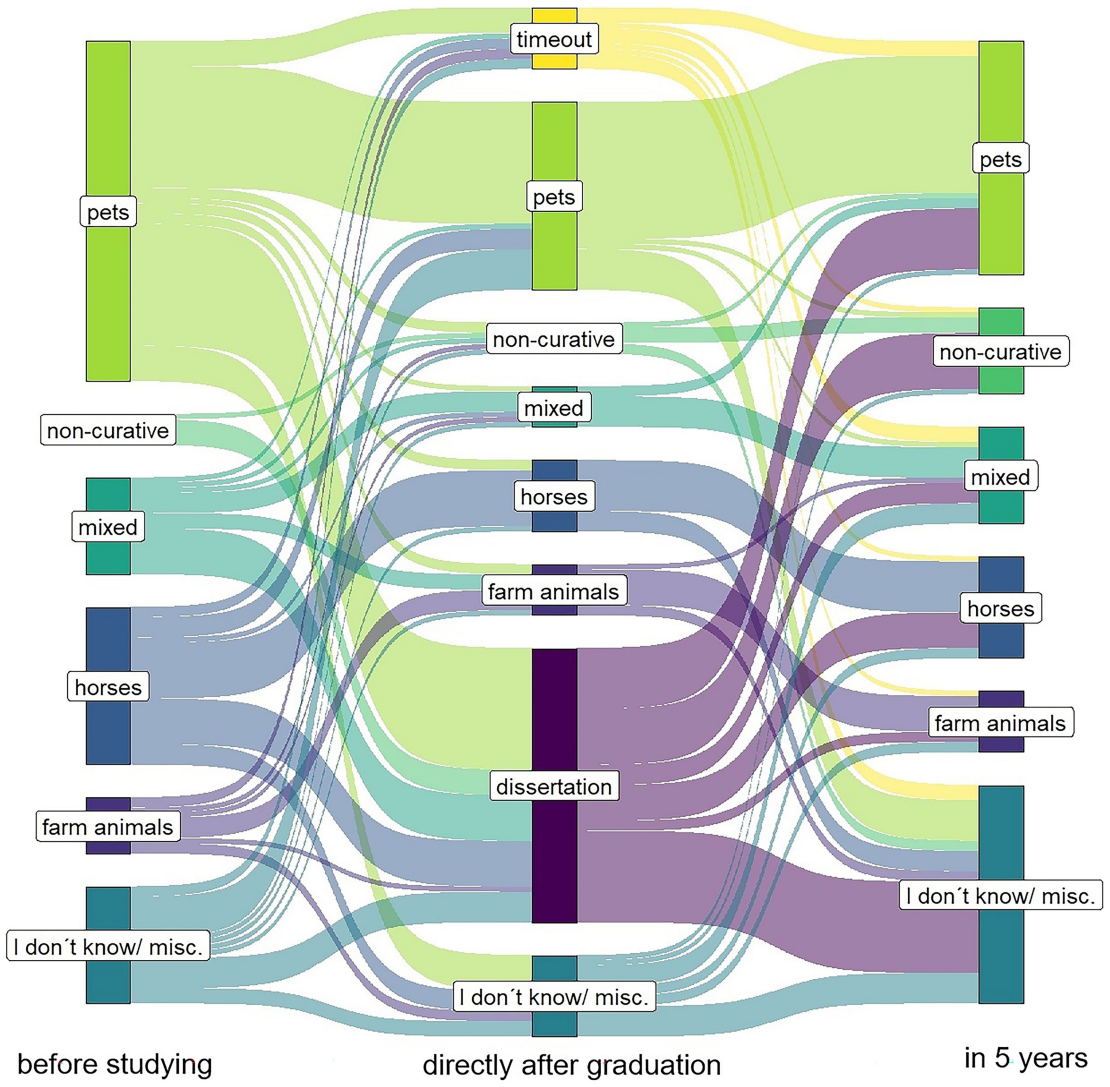
Decision movement showing which field veterinary graduates would like to work in before graduation, after graduation, and in 5 years’ time based on the results of a survey conducted among German veterinary graduates from the class of 2023.

Table 4 shows that pets were also the most strongly represented option before the studies. When looking at Tables 3, 4, a similar distribution between the veterinary fields can be seen both before the study and after the study and in 5 years’ time. However, Figure 2 clearly shows a decision movement of the individual participants from the desired veterinary field before their studies (left bar) to the plans after their studies (middle bar) to the field in which they see themselves in five’ time (right bar). Overall, 31% (*n* = 48) of respondents saw themselves in a different veterinary field in 5 years’ time than before their studies. Of those surveyed, 23% (*n* = 36) saw themselves in a certain field before their studies but did not yet know what they would like to do in 5 years’ time. Only 42% (*n* = 66) of participants wanted to work in the same veterinary field 5 years after graduation that they had aspired to at the beginning of their studies. Seven respondents (4%) did not know which veterinary field they would like to work in either before their studies or in 5 years’ time.

**Table 4:** Desired veterinary field in which the graduates wanted to work before their studies from a survey conducted among German veterinary graduates from the class of 2023.

Desired veterinary field before their studies	*n*	%
Pets	64	41
Ruminants	10	6
Horses	31	20
Pigs	1	1
Poultry	0	0
Exotic animals	3	2
Mixed	19	12
Non-curative	6	4
I did not have any preferences	21	13
Other	2	1
Total	157	100

### 3.4 Factors influencing the choice of veterinary field

Neither gender nor origin was associated with the field the participants would like to work in before their studies and in 5 years’ time (Tables 5, 6). Although the expected frequency (expected frequency = 1.2, actual frequency = 3) gave an indication that men were more likely to choose farm animals than women before studying, such an indication did not exist in 5 years’ time. It is noticeable that people who had kept horses before their studies were significantly less likely to choose working with pets and significantly more likely to choose working with horses both before their studies (*p* = <0.0001) and in 5 years’ time (*p* = 0.0010). Participants who had kept farm animals before their studies were significantly less likely to work in small animal medicine (pets) and more likely to work in a mixed-practice or non-curative fields in 5 years’ time than those who had not had farm animals (*p* = 0.0017). In contrast, there was no significant association between keeping farm animals before studying and future plans before studying (*p* = 0.3152). However, the expected frequency gave an indication that respondents who had kept farm animals before their studies saw themselves more frequently in farm animal medicine than those who did not have farm animals (expected frequency = 2.2, actual frequency = 5). Participants who had only kept pets before their studies saw themselves significantly more often working in small animal medicine (pets) and significantly less often in equine medicine both before their studies (*p* = <0.0001) and after 5 years (*p* = 0.0001) than those who had also kept other types of animals.

**Table 5:** Influence of gender, origin, and pets on future plans before studying from a survey conducted among German veterinary graduates from the class of 2023 (expected frequency in brackets)*.

			Pets	Horses	Farm animals	Mixed	Non-curative	I do not know	Other	Total	Fisher’s exact test
Gender	Male		6 (7.4)	4 (3.5)	3 (1.2)	1 (2.1)	1 (0.7)	0	0	15	
	Female		60 (58.6)	27 (27.5)	8 (9.8)	18 (16.9)	5 (5.3)	21	2	141	
	Total		66	31	11	19	6	21	2	156	0.3152
Origin	Rural		40 (37.5)	15 (17.6)	8 (6.3)	11 (10.8)	1 (2.8)	11	0	86	
	Urban		26 (28.5)	16 (13.4)	3 (4.8)	8 (8.2)	4 (2.2)	10	2	69	
	Total		66	31	11	19	5	21	2	155	0.2889
Animals at home	Just pets	Yes	53 (40)	8 (18.5)	4 (6.6)	10 (11.3)	5 (3.6)	9	2	91	
		No	14 (27)	23 (12.5)	7 (4.4)	9 (7.7)	1 (2.4)	12	0	66	
		Total	67	31	11	19	6	21	2	157	<0.0001
	Horses	Yes	4 (16)	20 (7.4)	3 (2.6)	5 (4.5)	0 (1.4)	8	0	40	
		No	63 (51)	11 (23.6)	8 (8.4)	14 (14.5)	6 (4.6)	13	2	117	
		Total	67	31	11	19	6	21	2	157	<0.0001
	Farm animals	Yes	9 (13.5)	7 (6.2)	5 (2.2)	5 (3.8)	1 (1.2)	7	0	34	
		No	58 (53.5)	24 (24.8)	6 (8.9)	14 (15.2)	5 (4.8)	14	2	123	
		Total	67	31	11	19	6	21	2	157	0.1258

* The variables ‘I do not know’ and ‘other’ were not included to calculate the expected frequencies, chi-square test, or the Fisher’s exact test.

**Table 6:** Influence of gender, origin, and pets on the decision of future plans in 5 years’ time from a survey conducted among German veterinary graduates from the class of 2023 (expected frequency in brackets)*.

			Pets	Horses	Farm animals	Mixed	Non-curative	I do not know	Other	Total	Fisher’s exact test/chi-square test
Gender	Male		4 (4.4)	2 (1.9)	1 (1.2)	2 (1.8)	2 (1.7)	4	0	15	
	Female		41 (40.6)	18 (18.1)	11 (10.8)	17 (17.2)	15 (15.3)	36	3	141	
	Total		45	20	12	19	17	40	3	156	0.9861
Origin	Rural		23 (26.6)	12 (11.8)	8 (7.1)	14 (11.2)	9 (9.4)	19	1	86	
	Urban		22 (18.5)	8 (8.2)	4 (4.9)	5 (7.8)	7 (6.6)	21	2	69	
	Total		45	20	12	19	17	40	3	155	0.5250 **
Animals at home	Just pets	Yes	38 (26.2)	6 (11.4)	4 (6.8)	8 (10.8)	9 (9.7)	24	2	91	
		No	8 (19.8)	14 (8.6)	8 (5.2)	11 (8.2)	8 (7.3)	16	1	66	
		Total	46	20	12	19	17	40	3	157	0.0001 **
	Horses	Yes	5 (11.7)	12 (5.1)	4 (3.1)	5 (4.8)	3 (4.3)	10	1	40	
		No	41 (34.3)	8 (14.9)	8 (8.9)	14 (14.2)	14 (12.7)	30	2	117	
		Total	46	20	12	19	17	40	3	157	0.0010
	Farm animals	Yes	3 (11.3)	6 (4.9)	4 (2.9)	8 (4.7)	7 (4.2)	6	0	34	
		No	43 (34.7)	14 (15.1)	8 (9.1)	11 (14.3)	10 (12.8)	34	3	123	
		Total	46	20	12	19	17	40	3	157	0.0017

* The variables ‘I do not know’ and ‘other’ were not included to calculate the expected frequencies, chi-square test, or the Fisher’s exact test.

** chi-square test.

### 3.5 Influence of the COVID-19 pandemic

It should be emphasized that those graduating in 2023 received practical training during the COVID-19 pandemic. Most of the participants (76%, *n* = 118) stated that the COVID-19 pandemic had had a strong negative impact on their practical training (Table 7). Only 9% (*n* = 14) agreed (mostly) with the statement ‘My university provided substitute/replacement offers during the COVID-19 pandemic to catch up on practical training’.

**Table 7:** Level of agreement with statements about the impact of COVID-19 pandemic using a Likert scale from a survey conducted among German veterinary graduates from the class of 2023.

	‘The COVID-19 pandemic had a strong negative impact on my practical training.’		‘During the COVID-19 pandemic my university provided substitute programs to catch up on my practical training.’	
	*n*	%	*n*	%
I agree	88	57	3	2
I mostly agree	30	19	11	7
I partially agree	26	17	50	33
I hardly agree	9	6	58	38
I disagree	2	1	29	19
Total	155	100	151	100

## 4 Discussion

This study provides initial insights into estimating how many veterinarians are entering which field of the veterinary market. It is surprising that only 42% of the participants wanted to work in the field they had aspired to before their studies. In addition, it was found that neither gender nor origin (urban/rural) was significantly associated with the choice of veterinary field.

### 4.1 Limitations

One limitation of this study was the cross-sectional cohort study design. However, it made it possible to gain a good insight into the graduate year of 2023, although future plans may differ from year to year. It should be particularly emphasized that the surveyed graduates had received practical training during the COVID-19 pandemic. It can be assumed that this influenced the graduates’ responses. Furthermore, the answers to questions about the past were based on memories and could therefore be influenced by their own perception (memory bias). Finally, it is important to bear in mind that due to the relatively small number of respondents, sometimes the subgroups consisted of only a few people, which weakened the significance of correlations between different factors.

The results of the future plans are only applicable to Germany, as (practical) training in other countries is structured differently. Nevertheless, the correlations between future and personal factors, for example, can provide indications for other countries. A systematic selection bias is not to be expected in this study as the topic was of equal interest to all members of the target population. This is also supported by the fact that the distribution between the universities was roughly homogeneous and the gender distribution among the participants corresponds to the gender distribution in the graduating year of 2023 (22).

### 4.2 Future plans directly after their studies and in 5 years’ time

The shortage of veterinarians in veterinary medicine is a much discussed international phenomenon (1, 6, 7, 9, 11, 14, 29). To properly assess and verify this phenomenon, among others, we need sufficient data from the veterinary profession and on the animal species that need to be treated.

This survey showed that almost half (48%, *n* = 76) of the participants would like to work in a practice after graduation and 15% more (63%, *n* = 98) 5 years later. This result is consistent with other studies (8, 17). It should be noted that, as the results of this study suggest, future plans of the participants often change during the undergraduate degree program. However, the results of this study are not sufficient to assess whether the number of graduating veterinarians planning to go into practice is enough to ensure adequate veterinary care. This requires further data from the veterinary profession and on the animal species that need to be treated. Various approaches have been developed in other countries to recognize shortages in veterinary care at an early stage (1, 30–32). Nonetheless, to date, in Germany, there is only one needs assessment study on one state and this only for livestock animals (8). In addition, there are veterinary statistics available from the Federal Veterinary Surgeons’ Association (Bundestierärztekammer) and the Veterinarian Atlas of Germany (Tierärzte Atlas Deutschland), which show how many veterinarians are practicing veterinary medicine in each federal state (22, 33). However, they do not publish any nationwide data, e.g., on actual working hours, the animals treated, and how many graduates enter practice after finishing their degree. This shows that in Germany there is currently not enough data to be able to properly assess the veterinarian shortage. Such data would help to develop well-founded and efficient solutions. Therefore, we see this study as a small contribution to this lack of data, taking a cohort of graduates into account.

### 4.3 Distribution of veterinary fields

The results show that most participants would like to work in a small animal field both before and after their studies and in 5 years’ time. This result is also reflected in other national and international studies in which small animal medicine was chosen most frequently after graduation (17, 18, 23, 34). Among other things, this can be explained by the fact that the prestige of an animal field, or the view of the field of work and everyday working life has a decisive influence on the choice of the future veterinary field (17–19, 35, 36). Dogs and cats are increasingly taking on the role of family members (37). Thus, small animal veterinarians are taking on the role of the friendly, empathetic doctor for family members. In society, the image of a veterinarian is dominated by the small animal sector or by the small animal veterinarian just described, and thus, it attracts especially those prospective students who find such an image appealing. As a result, those individuals are less able to identify with other fields (38). Although a large proportion of the participants wanted to work with small animals, it is not certain that this will meet demand. In fact, there are indications that there is also a shortage of veterinarians in small animal medicine (10, 39).

In contrast to small animal medicine, the prestige of farm animal medicine is not highly regarded among students (17). This could explain why graduates are significantly less likely to go into farm animal medicine than small animal medicine. Payne was able to explain this phenomenon in her study findings. To start a career in livestock medicine, students must have the feeling of ‘fitting in’ (40). That feeling was particularly lacking among women, marginalized ethnic groups, and those from the city or suburbs (41).

Another possible reason could be that students would like there to be more individual animal and intensive care in their future practical work (40). This type of practical work is reflected particularly in small animal and equine medicine. In contrast, the role of farm animal veterinarians has changed over the years (42–45). They are no longer concerned with just individual animals, but also with herd health, the food chain, and the commercial business (43, 44, 46). Consequently, the value of an individual farm animal could be considered less than the value of dogs, cats, or horses. Whether this view on farm animal medicine or the changing role of farm animal veterinarians influences career decisions after graduation has so far been little investigated and needs to be investigated in more detail in further studies.

### 4.4 Changes in future plans

When looking at the distributions of animal fields at the various points in time, it seems as if only a few changes occurred. However, in 5 years’ time, 54% (*n* = 84) of participants either saw themselves working in a different veterinary field than before graduation or they changed their original aspiration and did not yet know which sector they wanted to work in. This means that more than half of the participants changed their mind or were still undecided about their future plans. A previous German study also found that 49% of the surveyed students changed their career aspirations during their studies (16). In another German study, students even changed their mind in their first year of study (47). Similarly, in Croatia and England it has been shown that the preferred animal field changes over the course of studies (34, 48). These changes could be explained by the fact that there are many factors during the study program that influence students’ interests and future plans. Usko found that particularly internships and part-time jobs in a animal field have an influence on a graduate’s decision (16). A similar result was found in the USA. If students changed their desired field during their studies, they usually did so because new interests were sparked by veterinary courses (49, 50). Contact with practicing veterinarians, extramural internships, and practical experience also influence the choice of the animal field (5, 23, 40, 48).

Therefore, the degree program is an important phase of orientation. If the veterinary medicine course meant that students had to decide on a specific animal field at the beginning of their studies, it would no longer be possible for them to nurture an interest for other fields during their undergraduate studies. Instead, practical training in an animal field should be made as interesting as possible to attract students to this particular field (49). Therefore, it is up to educational institutions and lecturers for students to be exposed to every animal species if a shift of interest to another animal species is desired.

### 4.5 Factors influencing the choice of veterinary field

In the past, there have been calls to attract more male students to veterinary medicine to counteract the shortage of farm animal veterinarians (35, 51, 52). In fact, some studies even found that men preferred the livestock sector, and women were less likely to opt for farm animal medicine (17, 18). However, the results of this study question such claims, as there was no association between gender and the decision to go into (farm) animal medicine after graduation. This was also the case in other studies (16, 19). Although the expected frequency gave an indication that men are more likely go into farm animals than female before studying veterinary medicine, after graduation there was no such indication. It can be assumed that among other things the feminization of the veterinary profession in Germany is so far advanced (22, 53) that there are enough female role models in farm animal medicine and thus gender plays an increasingly rare role in decision-making regarding future plans. Nevertheless, it should be pointed out that even today gender discrimination still occurs in farm animal medicine and in rural regions (54) and could continue to result in women choosing farm animal medicine less often than men. However, this problem should not be solved by encouraging more men to enroll in veterinary programs, but by addressing gender discrimination.

Furthermore, in this study there was no association between the origin of participants and their choice of veterinary field directly after graduation and in 5 years’ time. However, this result conflicts with other studies (17, 19, 23, 36, 38). For example, graduates who grew up in rural areas chose to go into farm animal medicine more often than those who grew up in cities (17). Further studies are needed to investigate the connection between origin and the later choice of veterinary field. At the same time, it is interesting to note that the choice of future place of residence where the respondents want to live and work appears to be of secondary importance. Instead, just under half of the participants said that the place of work was not relevant as long as they were satisfied with the job (47%, *n* = 75). This result gives hope for believing that the shortage of veterinarians in rural areas, regardless of the type of animal treated, can be eliminated if the focus is on employee satisfaction and thus on working conditions (39, 55).

In this study it has been shown that there are significant associations between keeping certain animals and future career plans. This phenomenon was already shown in other studies (17, 18). It could be explained by the fact that students need contact with an animal species and the feeling of ‘fitting in’ before deciding to work with that kind of animal (40, 48). Contact with an animal and the feeling of ‘fitting in’ allows easy access to practical veterinary work and gives students more confidence if they also have in-depth knowledge of husbandry and behavior. Since only around 22% of the participants had contact with farm animals before their studies (Tables 5, 6), this shows a particular challenge for the training institutions; namely, if one wants to attract more people to a specific animal field, it is important to establish as many points of contact as possible so that students can develop a sense of ‘fitting in’.

## 5 Conclusion

The lack of knowledge about the current veterinary workforce and need for veterinarians carries the risk of misjudging a potential veterinarian shortage. We need reliable data that allow us to correctly assess a veterinarian shortage to forecast future scenarios. By capturing the future plans of German veterinary medical graduates of the class of 2023, this study forms a small component filling this gap.

An important finding of this study is that many veterinary medical students changed their mind during their studies or are unsure at the time of graduation where they would like to work after graduation. This should be taken into consideration when discussing implementing a study program where students have to choose an animal field at the beginning of their studies. Particularly noteworthy is the result that for almost half of the participants the place of work was not relevant as long as they were satisfied with their job. Regarding the preferred type of animal field, it was found that external factors, such as contact with an animal, influence the choice of field more than demographic factors such as gender or origin. It can be assumed that interest in a specific animal field starts through contact with individual animals. Therefore, it is likely that interest in certain fields can be encouraged by a wide range of offers at the universities. As comparatively few people had contact with farm animals before studying, there is an increased need for contact with farm animals during the undergraduate study program if it is wanted to attract more veterinarians to this field. These findings should be considered when discussing changing the admission requirements of universities of veterinary medicine to attract more people to a certain animal field or region.

Furthermore, longitudinal studies are needed, which record the career paths of veterinarians to document their distribution in the various veterinary fields, to recognize career changes, and investigate possible influences on them over the years.

## Data Availability

The datasets presented in this study can be found in online repositories. The names of the repository/repositories and accession number(s) can be found at: https://box.fu-berlin.de/s/GbFC9AwCff8mZkt.
